# Accessing doctors at times of need–measuring the distance tolerance of rural residents for health-related travel

**DOI:** 10.1186/s12913-015-0880-6

**Published:** 2015-05-29

**Authors:** Matthew Richard McGrail, John Stirling Humphreys, Bernadette Ward

**Affiliations:** Centre of Research Excellence in Rural and Remote Primary Health Care, School of Rural Health, Monash University, Northways Road, Churchill, VIC 3842 Australia; Centre of Research Excellence in Rural and Remote Primary Health Care, School of Rural Health, Monash University, PO Box 666, Bendigo, VIC 3552 Australia

**Keywords:** Doctors, Access, Rural residents, Primary health care, Travel behaviour, Distance

## Abstract

**Background:**

Poor access to doctors at times of need remains a significant impediment to achieving good health for many rural residents. The two-step floating catchment area (2SFCA) method has emerged as a key tool for measuring healthcare access in rural areas. However, the choice of catchment size, a key component of the 2SFCA method, is problematic because little is known about the distance tolerance of rural residents for health-related travel. Our study sought new evidence to test the hypothesis that residents of sparsely settled rural areas are prepared to travel further than residents of closely settled rural areas when accessing primary health care at times of need.

**Methods:**

A questionnaire survey of residents in five small rural communities of Victoria and New South Wales in Australia was used. The two outcome measures were current travel time to visit their usual doctor and maximum time prepared to travel to visit a doctor, both for non-emergency care. Kaplan-Meier charts were used to compare the association between increased distance and decreased travel propensity for closely-settled and sparsely-settled areas, and ordinal multivariate regression models tested significance after controlling for health-related travel moderating factors and town clustering.

**Results:**

A total of 1079 questionnaires were completed with 363 from residents in closely-settled locations and 716 from residents in sparsely-settled areas. Residents of sparsely-settled communities travel, on average, 10 min further than residents of closely-settled communities (26.3 vs 16.9 min, p < 0.001), though this difference was not significant after controlling for town clustering. Differences were more apparent in terms of maximum time prepared to travel (54.1 vs 31.9 min, p < 0.001). Differences of maximum time remained significant after controlling for demographic and other constraints to access, such as transport availability or difficulties getting doctor appointments, as well as after controlling for town clustering and current travel times.

**Conclusions:**

Improved geographical access remains a key issue underpinning health policies designed to improve the provision of rural primary health care services. This study provides empirical evidence that travel behaviour should not be implicitly assumed constant amongst rural populations when modelling access through methods like the 2SFCA.

## Background

In accord with the declaration of Alma Ata, ensuring adequate access to primary health care (PHC) services is vitally important for governments and health authorities of most countries [1, 2]. Good access increases timely utilisation of health services [3–5]. In contrast, populations without adequate access exhibit poorer health outcomes [6–9]. While access is a complex concept [10, 11], for health care consumers living in rural areas a key component of good access to health services is minimising the geographical barriers of distance and isolation, particularly when rural residents are required to travel outside of their immediate town to access health care.

Many small rural towns lack adequate resident health services, including doctors [12]. Despite numerous incentive policies offered by governments of many countries for some time, the recruitment and retention of doctors in small, often isolated, rural communities remains difficult [13–15]. This persistent problem reflects many professional and personal factors facing doctors who work and live in rural communities, including ‘unsociable’ working conditions characterised by longer hours, particularly on-call or after hours; difficulties associated with professional isolation such as taking leave from work, a lack or peer support, and limited access to professional development; and disinterest in living and raising their family in a rural area, often due to lack of employment opportunities for professional partners or poorer education choices for school-aged children [13, 16–19].

There exists a vast literature on access barriers to health and medical care in rural areas, with geographical isolation and distance foremost. Moreover, research has demonstrated associations between increased geographical barriers and decreased utilisation of inpatient, screening, outpatient and other community-based services [20–24]. Whilst many studies suggest that individuals in more remote setting accept increased travel as a routine part of their lives [25, 26], few have specifically investigated the travel behaviour of rural residents in relation to geographic barriers when accessing their usual PHC service [27–29]. Furthermore, little is known about ‘how far is too far?’ given the need for many rural residents to travel outside of their community to access a doctor [30]. This paper investigates self-reported health-related travel behaviour, both current or ‘normal’ travel time and maximum or upper tolerance travel time of residents in small rural communities when accessing a general practitioner (GP) for non-emergency PHC. We hypothesise that residents of sparsely settled rural areas are prepared to travel further than residents of closely settled rural areas given a need to access PHC services.

This empirical investigation of health-related travel behaviour of rural residents is very important for both health policy and service planning. Recently, the two-step floating catchment area (2SFCA) method has emerged as a key tool for service planners to identify rural access differences of PHC or define PHC shortage areas [31–34]. Critically, access models such as the 2SFCA method are dependent on the choice of catchment size(s) and related decisions on distance-decay functions [2, 35]. There is little information to guide which is the most appropriate function, such that Wang (2012) recently synthesised six different distance-decay functions to be effective [36]. Moreover, it is not known whether the same distance behaviour (that is, function homogeneity) applies equally to all rural populations, or whether residents of more sparsely-populated rural areas behave differently from those residents in more closely-settled regions.

## Methods

Five small rural communities within the states of Victoria and New South Wales in Australia were surveyed. Two communities were in closely-settled regions – that is, where the population density was 4–8 per square kilometre and three communities were in sparsely-settled regions – that is, where population density was only 0.5–1.0 per square kilometre [37]. Residents from the closely-settled rural communities need to travel on average 30 km to nearby alternative GPs, and these communities are located within the “Inner Regional” category of the Australian Standard Geographic Classification – Remoteness Areas (ASGC-RA) [38]. This compares to an average 60 km to an alternative GP for residents of the sparsely-settled rural communities which fall within ASGC-RA “Outer Regional”. The ASGC-RA classification has four rural categories, with “Inner Regional” covering 4 % of the area but 19 % of Australia’s population, “Outer Regional” 12 % of the area and 9 % of Australia’s population, and “Remote” or “Very Remote” categories covering 84 % of Australia’s area and 2 % of the population. For cost and methodological reasons, this study did not cover the two remote categories.

Each of the five communities was selected on the basis of the following common characteristics: having at least one resident GP; having a residential population < 2500; not being located within 100 km of a large regional or metropolitan centre; being demographically similar to many other small rural communities in Australia; and being situated in a region where residents have a choice of accessing GPs from at least three neighbouring larger towns. This latter criterion was included to ensure that residents didn’t have only one alternative option, as occurs when a small rural community is located ‘at the end of the line’. Similarly, none of the chosen communities were located close (<100 km) to large regional or metropolitan centres where the dominant ‘pull’ from a big centre would likely bias the travel behaviour in only one direction. Three of the communities had populations between 500 and 1000 and two had populations between 1500 and 2000.

The data were obtained via a mailed reply-paid questionnaire survey undertaken in August-September 2012. The questionnaire was distributed to every household in each community using the Australia Post unaddressed mail service [39]. One member of each household aged 18 or more was invited to participate in the survey. To maximise response rates, extensive community publicity was undertaken via media outlets (radio and newspapers), community forums, health and community services, school newsletters, and retail outlets in each locality prior to the questionnaire distribution. Three weeks after the initial mail out, a reminder letter was sent to all households [40]. Extremely high costs of face-to-face or telephone interviews across these widely-dispersed populations precluded our use of these methods. Ethics approval was obtained from the Monash University Human Research Ethics Committee.

Similar to the study undertaken by Shannon in 1979 [27], two specific questions related to travel behaviour were employed. The first question, “How far (kilometres) and how long (minutes) do you normally travel to visit a doctor (GP)?” provided two measures of *current* travel time. The second question, “What is the maximum time (minutes) you are prepared to travel to see a GP (for something that wasn’t an emergency)?” ascertained a measure of *maximum* travel time. Our questionnaire additionally included questions relating to other potential explanatory factors of travel behaviour including demographic characteristics and other potential predisposing factors such as poorer mobility or availability.

Means, percentiles and Kaplan-Meier survival functions were used to measure the relationship between increased distance and decreased travel propensity (that is, distance-decay). Significant differences by study group (closely- or sparsely-settled) were calculated using the logrank test. Bivariate associations of travel behaviour were calculated between potential explanatory factors and study group using median time and Mann-Whitney’s rank sum test. Most participants rounded their responses to the nearest 5–10 min (or kilometres). For this reason, ordinal multivariate regression models, with dependent time measurements in 10 min groupings, were calculated to measure the association between distance-decay and study group. These models adjusted for potential explanatory factors including two age groupings (<65 vs > =65), gender, employment status, occupational category (farmer, professional, other), current self-reported health status (5 levels from excellent through to poor), number of years at current residence, and several other access constraints including ratio of adults to cars being >1 at the residence, dependence on other family, friends or public transport services for transport support, not having a ‘usual’ doctor (GP) and greater length of time since last visit to a GP. Additionally, respondents indicated whether they had experienced a recent delay in accessing the doctor because of difficulty in getting an appointment, or more generally if access was ever a problem. All regression models were adjusted for clustering by town, where observations within the five collection towns may not be independent. All calculations were performed using StataSE 12 (StataCorp, College Station, TX, USA) and a significance level of 5 % was used throughout.

## Results

A total of 1079 questionnaires were completed, yielding an overall response rate of 26 % (ranging between 23 and 34 % across the five communities). Though this rate was disappointing, our sample captures the behaviour of those residents most concerned with, or likely to need, the services of a GP. Of the 1079 responses, 363 were from residents in closely-settled areas and 716 were from residents in sparsely-settled areas. Based on the demography of the most recent 2011 census, females (68 %) were over-represented in each community surveyed. Respondents aged under 45 (13 %) were under-represented, but those in the 45–64 and 65+ age groups were evenly split. Notably, there were no differences in the observable characteristics of the two study groups across all enabling and need factors (age, gender, paid employment, self-reported health and last utilisation time), thus concerns of low response rates are minimised for the purpose of this study.

**Fig. 1:**
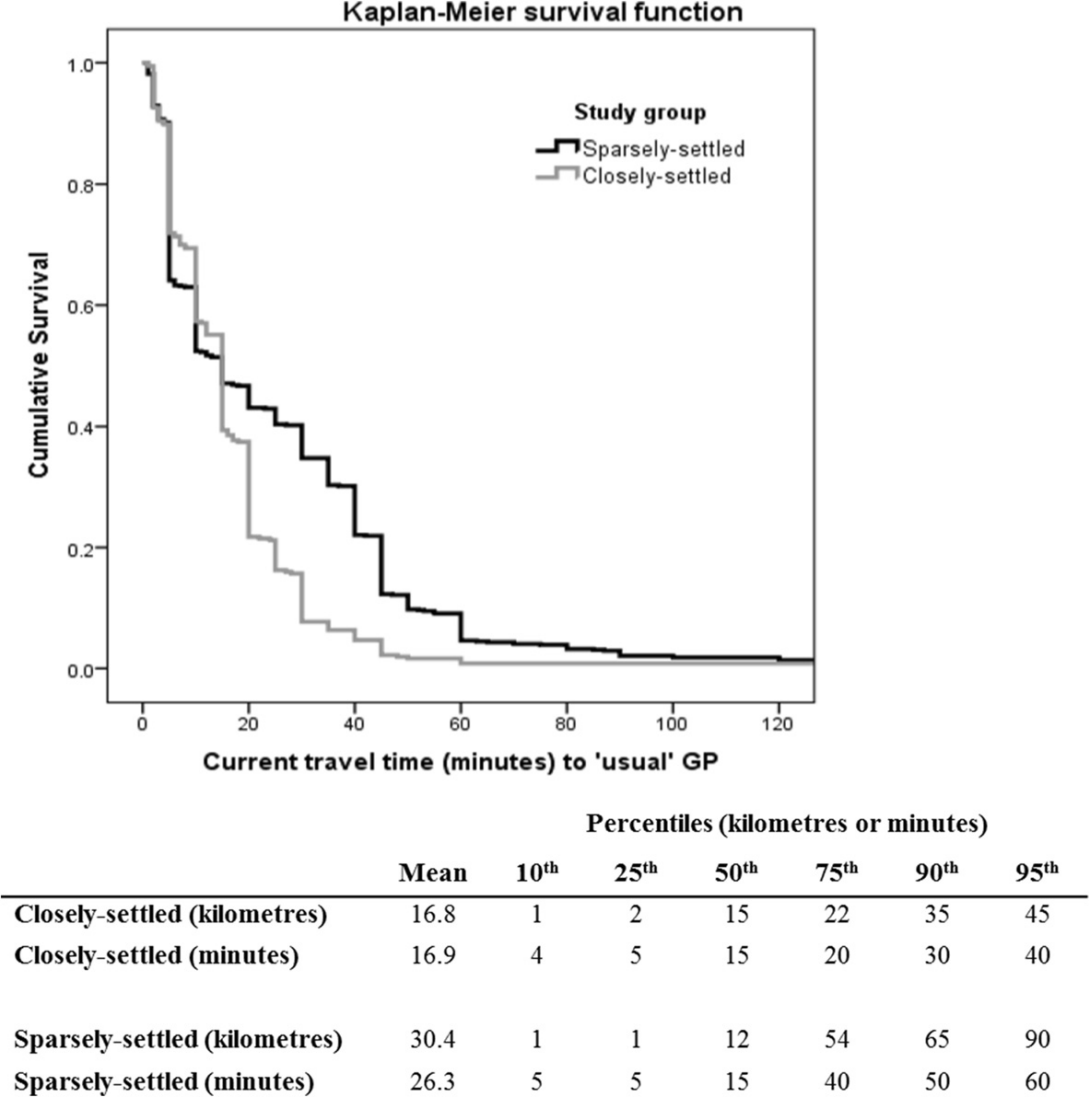
Distance-decay of resident’s current travel time to access their ‘usual’ doctor (GP) for non-emergency care

Figure 1 shows the difference between residents from closely- and sparsely-settled communities in *current* travel behaviour to their ‘usual’ doctor (GP). The current travel behaviour for almost half of the residents of both sparsely- and closely-settled communities is nearly identical since similar proportions do not travel outside of their town. However, for the remaining half who either choose to travel outside of their community or are unable to access services locally, there is a significant difference in their travel distance (p < 0.001). Residents of the sparsely-settled communities travel, on average, about 10 min further than closely-settled communities (26.3 min vs 16.9 min), and these differences are about +20 min for the 75^th^, 90^th^ and 95^th^ percentiles (see Fig. 1).

**Fig. 2:**
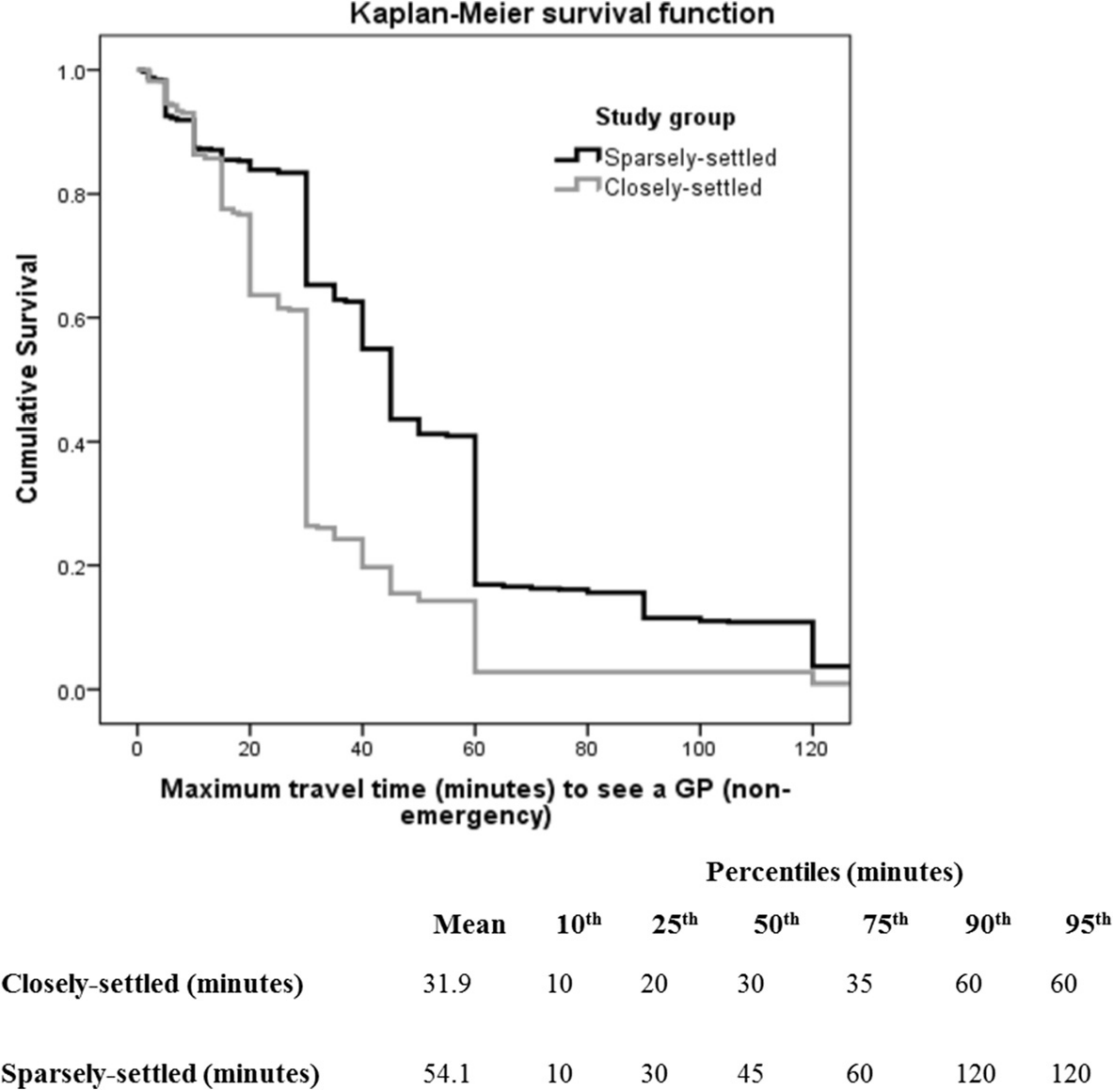
Distance-decay of resident’s maximum time prepared to travel to access a doctor (GP) in a non-emergency

Differences between our two study groups in terms of the ‘*maximum’* time they were prepared to travel were even more apparent, with closely-settled populations willing to travel, on average, only 31.9 min to access a GP compared to 54.1 min for sparsely-settled populations (Fig. 2). In addition, the two survival functions are much quicker to separate (from about p = 0.85, vertical axis) compared to those in Fig. 1 (from about 0.50). The clearer distance between the two survival curves indicates a stronger effect size of community type on maximum time willing to travel (p < 0.001). Notably, only about 25 % of closely-settled residents are prepared to travel more than 30 min to see their GP, while the equivalent value for sparsely-settled residents is around 65 %. Moreover, 41 % of sparsely-settled residents are prepared to travel at least 60 min, and about 15 % are prepared to travel 120 min, but only 3 % of closely-settled residents are willing to travel more than 60 min.

The bivariate analyses between all potential explanatory factors and travel behaviour are shown in Table 1. Notably, only gender, self-reported health and years at current residence were not significantly associated with travel behaviour and are dropped from further multivariate analysis. Many of the factors are strongly associated (p < 0.001) with increased travel, including currently being in paid employment, aged under 65, having to rely on others for transport support and experiencing general access problems or difficulties getting an appointment. Additionally, increased travel was strongly associated with not having a usual GP and residing in a household with fewer cars than adults.

**Table 1:** Bivariate associations of potential access explanatory factors with health-related travel time

	Sub-group	n (%)	Current travel (minutes – median)	p-value	Maximum travel threshold (minutes – median)	p-value
Age	<65	580 (54 %)	15	0.251	40	<0.001
	> = 65	497 (46 %)	10	-	30	-
Gender	Male	344 (32 %)	15	0.899	35	0.992
	Female	733 (68 %)	15	-	35	-
Currently in paid work	Yes	469 (44 %)	20	<0.001	40	<0.001
	No	588 (56 %	10	-	30	-
Years at current residence	<5 years	204 (19 %)	10	0.110	40	0.654
	> = 5 years	867 (81 %)	15	-	35	-
Ratio of household cars to adults	<1	248 (23 %)	10	<0.001	30	0.117
	> = 1	831 (77 %)	15	-	35	-
Rely on others for transport support	Yes	88 (8 %)	30	<0.001	50	0.001
	No	987 (92 %)	15	-	35	-
Access ever a problem	Yes	462 (43 %)	20	<0.001	45	<0.001
	No	612 (57 %)	10	-	30	-
Delayed access – difficulty getting appointment	Yes	356 (33 %)	20	<0.001	45	<0.001
	No	717 (67 %)	10	-	30	-
Don’t have a ‘usual’ GP	Yes	67 (6 %)	40	<0.001	45	0.001
	No	1012 (94 %)	15	-	35	-
Last visit GP	>3 months	223 (21 %)	15	0.055	35	0.014
	<=3 months	855 (79 %)	15	-	40	-
Self rated health	Poor-Fair	244 (23 %)	15	0.828	40	0.696
	Very good	818 (77 %)	15	-	35	-

Table 2 shows the multivariate analyses of strength of association between travel time (both *current* and *maximum prepared to travel*) and study group, whilst controlling for a range of sentinel demographic and access-related covariates, as well as the clustering town effect. Model 1 shows that the odds ratio (OR) of currently travelling longer (where each ordinal step represents 10 min) for residents of sparsely-settled communities compared to residents of closely-settled communities is above 1.0 but not statistically significant (OR 1.41, 95 % CI 0.44–4.75). The wide confidence interval is due to the reduced power after accounting for town clustering. Other factors which are significantly associated with increased current travel include being in paid work (OR 1.68, 95 % CI 1.40–2.01), reliance on others for transport (OR 2.92, 95 % CI 1.89–4.54), having experienced some access problems (OR 1.66, 95 % CI 1.08–2.55), not having a usual GP (OR 2.88, 95 % CI 2.03–4.08) and delaying access due to difficulty of getting an appointment (OR 1.48, 95 % CI 1.06–2.07) thereby necessitating travel to more distant services. Reduced travel times were associated with households with less than one car per adult (OR 0.59, 95 % CI 0.37–0.94).

**Table 2:** Multivariate ordinal regression models of current and maximum travel time to access a doctor (GP)

	Model 1: Current travel time		Model 2: Maximum prepared travel time		Model 3: Maximum prepared travel time (accounting for current travel time)	
	OR, 95 % CI	p-value	OR, 95 % CI	p-value	OR, 95 % CI	p-value
Reside in sparsely-settled area	1.44 (0.44–4.75)	0.551	3.62 (1.81–7.27)	<0.001	2.68 (1.78–4.03)	<0.001
Currently in paid work	1.68 (1.40–2.01)	<0.001	1.34 (1.18–1.52)	<0.001	1.11 (0.91–1.36)	0.301
Ratio of household cars to adults <1	0.59 (0.37–0.94)	0.026	0.77 (0.51–1.16)	0.212	0.95 (0.76–1.17)	0.612
Rely on others for transport support	2.81 (1.45–5.45)	0.002	2.20 (1.85–2.63)	<0.001	1.23 (0.80–1.91)	0.341
Access ever a problem	1.66 (1.08–2.55)	0.021	1.16 (0.79–1.68)	0.449	1.00 (0.72–1.38)	0.987
Delayed access – difficulty getting appointment	1.48 (1.06–2.07)	0.023	1.45 (1.07–1.96)	0.018	1.29 (1.03–1.60)	0.024
Age < 65	0.86 (0.67–1.11)	0.243	1.45 (1.06–1.97)	0.019	1.72 (1.34–2.20)	<0.001
Don’t have a ‘usual’ GP	2.88 (2.03–4.08)	<0.001	1.81 (1.09–2.99)	0.021	1.11 (0.73–1.68)	0.639
Current travel time	-	N/A	-	N/A	1.71 (1.46–1.99)	<0.001

*OR* Odds ratio; *CI* Confidence interval

Similar patterns are seen in Model 2 for maximum travel time, with one notable difference between study groups (Table 2). The odds ratio of being prepared to travel longer for residents of sparsely-settled communities is large and statistically significant (OR 3.62, 95 % CI 1.81–7.27), compared to residents of closely-settled communities. Poor access to vehicles and experience of some access problems are not significantly associated with different maximum travel times, while respondents aged <65 are prepared to travel further (OR 1.45, 95 % CI 1.06–1.97). Respondents who delayed access due to experiencing difficulty getting an appointment (OR 1.45, 95 % CI 1.07–1.96), who don’t have a usual GP (OR 1.81, 95 % CI 1.09–2.99) and who relied on others for transport (OR 2.20, 95 % CI 1.85–2.63) were associated with greater maximum travel times.

Model 3 includes the same factors as Model 2, with the addition of current travel time (i.e. the outcome of Model 1). Notably, the odds ratio of being prepared to travel longer for residents of sparsely-settled communities remains significant (OR 2.68, 95 % CI 1.78–4.03), compared to residents of closely-settled communities, even after accounting for both town clustering and current travel behaviour. The inclusion of current travel behaviour has removed the significance of being in paid work, relying on transport support and not having a usual GP – which were the strongest factors associated with current travel times in Model 1.

## Discussion

Geographical access remains a key determinant of health service utilisation at times of need, so it is important that health service planners minimise access barriers in relation to the provision of PHC services. To assist them, it is vital to understand rural resident’s travel patterns or preferences when accessing PHC, as well as the effect of any individual-level or service-level constraints. In view of the need for evidence-based policy, the findings from this study of rural healthcare-seeking travel behaviour provide a significant addition to the literature.

Our hypothesis that residents of sparsely-settled communities are prepared to travel significantly further to access a GP for non-emergency utilisation compared with residents of closely-settled rural communities was confirmed. Put another way, closely-settled residents are less prepared to travel as far as sparsely-settled residents for non-emergency health care. This finding is important to health service planners in obtaining a clearer picture of rural access and identifying rural communities with problematic access. Modelling of population-level access, notably with the 2SFCA method, is intended to ‘match’ the behaviour of the population. All previous applications of the 2SFCA method implicitly assume catchment sizes are unchanged for all rural populations. Our study provides important new empirical evidence that catchment sizes and related distance-decay functions should not be identical when modelling access across large rural areas such as tested here in two states of Australia. That is, distance tolerance changes in different rural contexts.

While current travel times and distance were higher for residents in sparsely-settled areas, the overall effect size, compared to closely-settled, was considerably lower than for the maximum distance they were prepared to travel. Additionally, allowance for clustering on the five selected towns removed the significance of the longer current travel distance characterising sparsely-settled areas. The main problem with using existing travel patterns as a measure of typical (potential) travel behaviour in rural areas is that it can be strongly influenced by local geography and current availability of existing services. We were very deliberate in our selection of communities to ensure that each had a range of access options, including at least three alternative rural communities nearby while not falling within the shadow of a dominant (large) metropolitan city. Moreover, local availability was similarly distributed with about 45 % of residents accessing services within their community in both types of towns, thus our study’s current travel time differences were not biased by the number of residents travelling out of their town to access a GP.

A much stronger (and statistically significant) association between travel behaviour and type of rural location is seen when measuring the maximum distance/time residents are prepared to travel. Unlike current travel time, maximum time residents are prepared to travel is less likely to be conditioned by availability. This was confirmed in Model 3, where the addition of current travel time (which is strongly linked to availability) did not remove the significance of sparse-area residents having a higher distance tolerance. Whilst residents who experience very good within-town access will expect to have significantly lower current travel times than residents with poor within-town access, the maximum travel times of residents in these two scenarios is unlikely to be greatly affected by service availability. This pattern is notable in Fig. 2, with sparsely-settled residents consistently more likely to accept travelling up to 20, 30, 45, 60 and even 120 min to see a GP (for non-emergency care).

Key parameters required for population-level access models, like the 2SFCA method, are the related catchment sizes and distance-decay functions [31, 35, 41, 42]. To date, however, application of these models by health service planners, especially in rural areas, is limited by the absence of evidence to justify the choice of catchment sizes and distance-decay functions. Our study provides empirical data, from an Australian geographical context, to guide these choices. Firstly, our data indicate that the upper boundary of the access catchment should be lower in closely-settled areas, with 40–60 min being appropriate compared with 80–120 min in sparsely-settled areas. Models which use a single catchment size for all rural areas are likely to be inappropriate for some settings. Secondly, the distance-decay function which defines how likely residents are to tolerate accessing services at specific distance barriers is similarly shaped but steeper in its decay in closely-settled compared to sparsely-settled rural areas. Our data suggest that a step decay function such as in Luo’s Enhanced 2SFCA [42] with only three steps is likely to be too crude, but the exact function choice is still not clear.

Differences in geography were not the only explanatory factors accounting for differences of travel behaviour. Other constraints also influenced behaviour with respect to accessing medical care. Younger residents and those currently employed were prepared to travel further, reflecting their higher mobility and wider activity area outside of their immediate community. Similarly, residents with ready access to vehicles were more likely to travel further. Residents who have experienced either a recent delay in access due to difficulty in getting an appointment or other general access problems, who don’t have a usual GP or who are dependent on others for transport were also more likely to travel further, probably because of necessity rather than choice. Notably, our multivariate model demonstrated that residents’ current location type remains a significant determinant of travel behaviour (distance tolerance) differences even after adjusting for other access factors.

The results from this study should be used with caution as they are generalisable only to rural places exhibiting similar geographies. In the case of Australia, where these rural places include the many small communities located throughout the broad-acre agricultural regions of the country, the results still have broad application to health service planners responsible for modelling access patterns of rural residents. In contrast, residents of metropolitan areas and large regional centres are unlikely to access services outside of their immediate location since there are many options within these large populated areas, so that more distant services located in smaller surrounding communities are unlikely to be attractive alternatives. Residents of highly isolated or ‘end of the line’ rural communities require significant effort to reach alternative access options, so that this group is also more likely to accept the need to travel large distances to health care at times of need, though the specifications of their travel behaviour will be largely dependent on the location of their next nearest service centre.

As previously raised, the low response rate for the survey is a limitation of this study. This outcome is typical of broad questionnaire surveys, with increased survey response rates in geographically-dispersed rural communities requiring significantly more resources. We acknowledge that delivery-and-collection questionnaires or interviews, either face-to-face or computer-aided telephone interviews, generate higher response rates, but our mailout methodology with extensive community publicity was employed because of the very high costs associated with using alternative survey methods for the dispersed populations comprising this study. However, it should be noted that our two study groups were equally matched on the key demographic factors. In addition, the under-representation of males and persons aged less than 45 years potentially biases our results, although these groups are typically the lowest users of GP services [43]. Moreover, while every attempt was made to ensure the communities were ‘typical’ of their settlement areas, it is possible that some specifically ‘local’ issues unknown to us may have skewed the results. A larger sample of different communities would reduce the likelihood of this outcome, but resource limitations and the logistics associated with studying large numbers of widespread rural communities for this study precluded this approach. These limitations notwithstanding, this research has generated new insights to how travel behaviour changes in different rural contexts.

## Conclusion

Access remains a key issue underpinning the provision of primary health care services in rural areas. Hence it is imperative that assumptions within service provision models are underpinned by evidence. This study provides important new empirical evidence showing that travel behaviour, a key factor in modelling access, should not be modelled as being constant amongst all rural populations. Indeed, the differences which residents are prepared to travel for medical care at times of need, differ significantly between sparsely-settled and more closely-settled rural communities. Accordingly, access modelling must account for these different travel thresholds and distance-decay when setting the underlying parameters. Failure to do so can result in problematic model outcomes which are misleading to the end user such as health service planners.
